# Artificial Intelligence Enhanced Content Review for Curriculum Evaluation in Nursing Education: A Pilot Study

**DOI:** 10.1097/NNE.0000000000002243

**Published:** 2026-07-09

**Authors:** Regina G. Russell, Md. Mashiur Rahman Khan, Lia Nagge, Hannah McGrew, Kendra Faucett, Xingchen Wei, Mary S. Dietrich, Julia Phillippi

**Affiliations:** **Author Affiliations:** School of Nursing, Vanderbilt University, Nashville, Tennessee.

**Keywords:** generative artificial intelligence, health equity, health professions education, nursing evaluation research, quality improvement

## Abstract

**Background:**

Curricular evaluation is core to nursing education and can benefit from artificial intelligence-based automation. Large language models offer new opportunities for scalable, structured document analysis.

**Purpose:**

We developed a rubric to identify elements related to health equity, including whether the content addresses vulnerable groups, social factors in health, and clinical applications.

**Methods:**

A chatbot using retrieval-augmented generation (RAG) was configured with structured rubric-based instructions. The reliability of chatbot ratings of 9 lecture slide sets from one nurse-midwifery course was compared to ratings from 3 faculty reviewers.

**Results:**

The chatbot achieved reliability in repeated ratings of 84% to 91% across assessed topics. Chatbot alignment with faculty raters across topics was more variable and ranged from 56% to 89% agreement.

**Conclusions:**

An RAG-based large language model system demonstrates promising reliability for automated curriculum screening. This tool can be used to assist, but not replace, human insights and summative judgments.

Curricular evaluation is a standard aspect of educational program implementation and improvement that may benefit from artificial intelligence (AI)-based automation. New computational tools can find and summarize patterns across extensive document stores with increasing accuracy. The integration of large language models (LLMs) into educational evaluation offers a novel approach to support data analysis, generate feedback summaries, and identify trends.^1–3^

By leveraging generative AI tools, nurse educators can streamline the evaluation process and inform curriculum improvement.^4,5^ When embedded within a structured feedback loop, these tools can enhance evaluators’ capacity to make data-informed decisions, ultimately contributing to more responsive and equitable nursing education practices.^6,7^ However, it is critical to assess the limitations of generative AI and the significant likelihood of error during use.^8^

One area of ongoing curricular interest for nursing education is health equity. According to the American Association of Colleges of Nursing Essential Competencies, health equity should be “deeply integrated” throughout the curriculum, including in simulations, interprofessional education, and community-focused curricula.^9^ The American College of Nurse-Midwives includes health equity as part of core professional responsibilities, requiring “advocacy for health equity, social justice, and ethical policies in health care.”^10^ Educators should equip students to recognize and address structural and social drivers of health through assessment, health promotion, education, collaboration, leadership, and advocacy.^9,10^ This project builds and tests an LLM system with custom instructions on the task of evaluating course content related to health equity.

## Defining Health Equity

A clear definition and description of the characteristics of health equity are important to guide education and evaluation since perspectives differ on meaning.^11^ Due to this complexity, it was crucial to establish a shared understanding of health equity before creating chatbot instructions.^11–12^ We used Lewis et al^11^ as our primary source for reviewing definitions of health equity and establishing our scoring rubric. Using this document and our knowledge of nursing education, we established 4 topic areas as essential curricular elements when teaching about health equity. These are coverage of health equity, antecedents to health equity, consideration of vulnerable groups, and clinical applications.

## Antecedents to Health Equity

Six major factors shaping health equity were described by Lewis et al^11^ in 2023. These antecedents span environmental, economic, political-legal, societal, research, and digital technology dimensions of equity. To be most effective, curricula that include objectives related to health equity need to address these diverse background factors.^13^

## Consideration of Vulnerable Groups

Vulnerability for people in healthcare exists at the intersection of risk and power.^14^ Examples of vulnerable groups include children and the elderly; racial, ethnic, sexual, gender, and religious minorities; and people who are disabled, pregnant, incarcerated, or low-income.^14,15^ In addition to the 6 antecedents, it is critical for nursing students to appreciate the impact of vulnerability on individuals seeking health care.

## Purpose

The purpose of this study was to use multiple methods to test the reliability, validity, and utility of a custom chatbot in providing automated feedback on health equity content in teaching presentations. The research was centered around a retrieval-augmented generation (RAG) architecture. RAG means users can attach content for the chatbot to use in producing outputs. For example, a chatbot with basic instructions about educational evaluation is given a rubric for scoring content. The user can then upload educational content for assessment. The use of an RAG chatbot system allows users to leverage existing institutional resources without major revisions to commercially developed LLMs.

Research steps included: (1) development of customized system architecture and instructions, (2) comparison of prompts and language models, (3) reliability testing of repeated outputs, (4) assessment of validity with expert raters, and (5) conduct of an internal focus group regarding utility. The work was designed to provide information useful in nursing and health professions education curricular design, revision, and evaluation. This study was reviewed by the appropriate institutional review board (IRB) and designated exempt research (IRB #240386).

## Methods

### Development of a Health Equity Rubric

Our research team developed a rubric to identify elements related to health equity in course PowerPoint slides from course lectures (without audio). The rubric included whether the content addressed antecedents to healthy equity, vulnerable groups, social factors in health, and clinical applications. The rubric was designed to guide the chatbot summaries and provide reliable output across a set of standard items. Chatbot custom instructions with the embedded health equity rubric are available under a noncommercial creative commons license (https://futureflo.vueinnovations.com/).

Each topic in the final rubric (see Supplemental Digital Content Appendix, Appendix A https://links.lww.com/NE/C370) was scored on a 3-point ordinal scale (1—low, 2—medium, and 3—high), and a corresponding narrative summary produced with reference to specified pages in the document. Key evaluation questions aligned with these 4 topics:

- Coverage of Health Equity: Are health equity principles or social determinants of health defined?
- Coverage of Health Equity Antecedents: Is there a mention of health equity antecedents, and to what extent are they explored?
- Addressing Vulnerable Groups: Is a vulnerable group(s) identified, and is there a discussion about the barriers they may face?
- Application of Health Equity in Clinical Practice: Are there any resources or steps provided to apply health equity concepts in clinical practice?

### Development of System Architecture and Instructions

An existing AI chatbot assistant, Future FLO,^8^ was customized to include the health equity rubric and output specifications. The chatbot is referred to as FLO25, or Feedback Loops and Outcomes 2025. An RAG architecture was selected to tie chatbot outputs to the actual lecture slide content, reducing unsupported or “hallucinated” responses.^16,17^

Figure 1 illustrates the system architecture used to evaluate instructional materials through the FLO25 chatbot. The workflow begins with 2 sources of information: (1) the foundational custom instructions with scoring rubric and (2) the uploaded PDFs of lecture slides. When the user inputs a specific task prompt, these sources of information are routed through a preprocessing pipeline that includes text extraction, cleaning, and chunking to prepare content for retrieval. All processed text is then made into an indexed database. During evaluation, the RAG-enabled FLO25 chatbot retrieves relevant lecture slide segments and applies the rubric and workflow instructions to generate output to address the task prompt. This output includes ratings, summaries, and page-referenced evidence.

**Figure 1.**
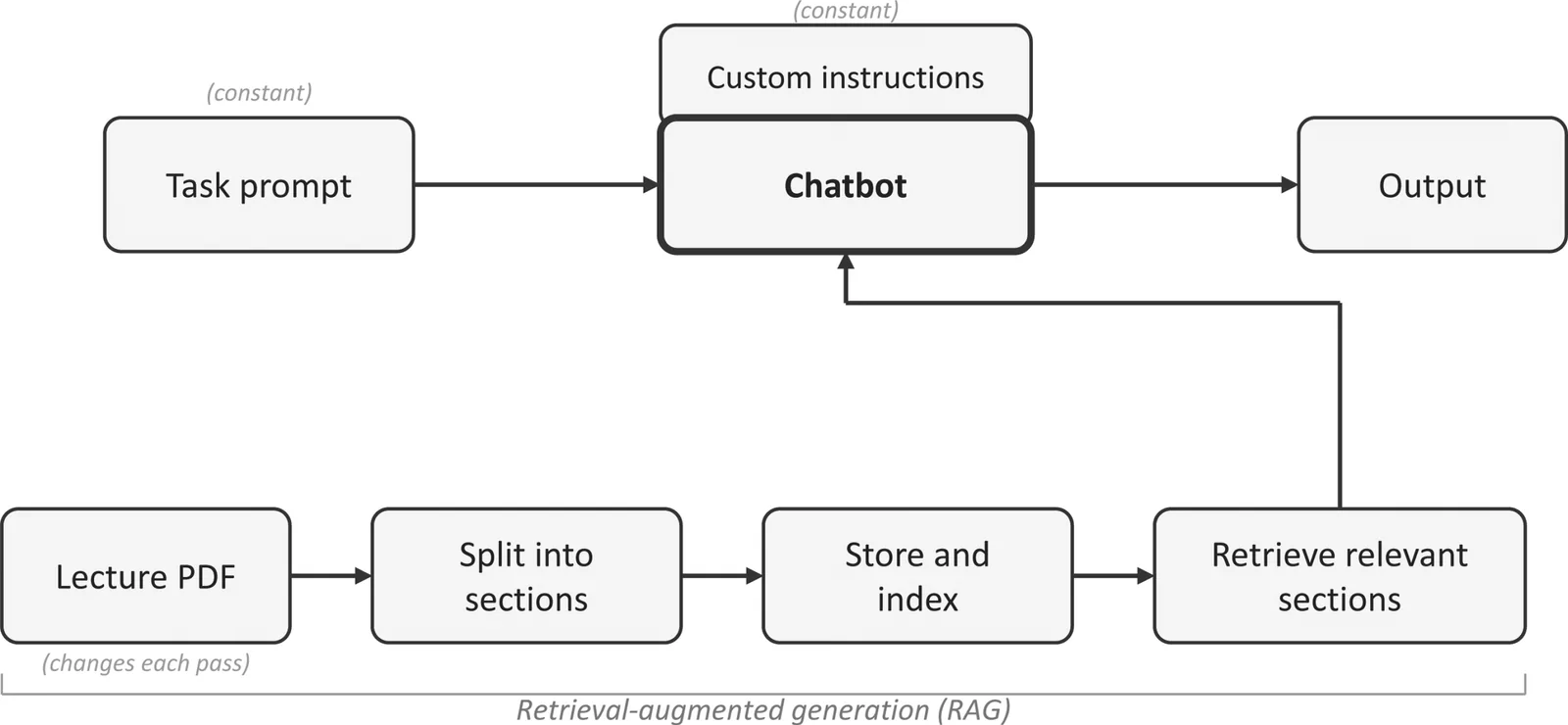
RAG chatbot architecture.

Chatbot custom instructions were written to ensure the AI-based assistant would respond as an evaluation assistant, prioritizing accuracy and precision in outputs.^8^ These instructions established the assistant’s primary topics for review, operational workflows, and output standards. To ensure the FLO25 chatbot could recognize key aspects of health equity, we inputted the rubric described earlier (Supplemental Digital Content Appendix, Appendix A https://links.lww.com/NE/C370).

### Selection of Large Language Model and Task Prompt

The team used Amplify,^18^ an enterprise-grade generative AI platform that provides access to several LLMs. We simultaneously tested 3 LLMs and 3 task prompts for use in this study. We adapted a published scoring tool for this purpose.^8^ The GPT-4,^19^ Claude 3.5 Sonnet V2,^20^ and Claude 3.7 Sonnet^21^ LLMs were tested and evaluated by the research team for use in this project. We wanted to select one LLM that provided quality output that faculty could use to make improvements to their lectures. In addition to selecting an LLM, we needed a task prompt to give the FLO chatbot to structure its outputs.

Task prompts determine the tone and level of detail of the outputs. Users input task prompts to tell the chatbot to perform a set of actions using the chatbot’s custom instructions and uploaded content. Three task prompts of varying specificity were tested across all 3 models.

Prompt 1 was a targeted query asking the system to identify specific antecedents to health disparities, including structural inequities, social determinants of health, and discrimination or bias, within lecture slides, with page-level citations. Prompt 2 asked the system to identify one major strength and one gap in the lecture’s health equity coverage with supporting reasoning. Prompt 3 instructed the system to follow its full custom instructions for the Health Equity Content Reviews workflow, working through each rubric element systematically to produce structured output suitable for faculty feedback.

Each prompt output from each LLM was rated by 3 authors on 4 dimensions: accuracy, completeness, alignment with the health equity rubric, and overall quality, using a 1 to 7 Likert scale (1 = very low, 7 = very high). Data were collected via a web-based survey form.^22^ Scores across the 4 dimensions were summed per prompt (maximum score = 28) and averaged across the 3 prompts to generate a single composite performance score per LLM. These composite scores informed the selection of Claude 3.7 Sonnet as the primary LLM and Prompt 3 as the task prompt.

### Retrieval-Augmented Generation

Nine PowerPoint slide sets (529 individual slides) covering different clinical content areas were selected from one nurse-midwifery course, *Care of the Newborn*. Each slide set was uploaded into the chatbot to be used in RAG, as described in the introduction. All content was evaluated by the FLO25 chatbot using Task Prompt 3, the full rubric-based structured review prompt selected by the process outlined above.

### Reliability Testing with Repeated Outputs

To assess output stability, FLO25 was run 15 times sequentially on each slide set using identical inputs and instructions, resulting in 135 total evaluation runs across the 9 lecture slide sets. Reliability was assessed for each of the 4 rubric topics independently. Exact interrun agreement was defined as the proportion of repeated runs in which the chatbot assigned the identical rating (low, medium, or high) for a given topic when presented with the same input. Agreement was calculated across the 15 repeated runs for each lecture slide set and then averaged across the 9 slide sets to produce a single topic-level reliability estimate.

### Validity Testing with Faculty Raters

Three faculty with expertise on the lecture content independently rated each PowerPoint slide set using the same health equity rubric provided to the chatbot. These raters were unaware of the chatbot’s reliability testing results. Alignment with expert scoring is a form of validity testing.^23^ The proportion of agreement between the modal chatbot rating for each slide set and the modal faculty rating for the respective slide set was used to assess criterion validity of the chatbot ratings with expert faculty ratings.

### Internal Focus Group Regarding Utility

A focus group script regarding the value and usefulness of this evaluation tool was developed using techniques described by Rubin and Rubin.^24^ The lead author facilitated a team discussion with all coauthors. A teleconferencing system^23^ was used to generate a meeting transcript, and a summary was produced by Claude 3.7 on the Amplify system and verified by 2 authors.

## Findings

### Comparison of Prompts and Language Models

Performance scores for 3 LLMs (GPT-4o, Claude 3.5 Sonnet V2, and Claude 3.7 Sonnet) were averaged across 3 evaluation prompts, each scored on accuracy, completeness, alignment, and quality (max score = 28). Claude 3.7 achieved the highest average score (23), outperforming both GPT-4o and Claude 3.5 (20 each). Prompt 3, with the full rubric review, provided the best potential feedback for educators.

### Reliability Testing With Repeated Outputs

Figure 2 displays the internal reliability of repeated scoring of the same slide set using the FLO25 chatbot. Interrun agreement was high across topics, exceeding 90% for Health Equity and Vulnerable Groups, and remaining above 80% for Antecedents and Clinical Applications. These findings suggest that the FLO25 chatbot produces stable categorical ratings with minimal variance when given identical inputs, supporting its reliability as an automated screening tool for curriculum evaluation. Supplemental Digital Content Appendix, Appendix B https://links.lww.com/NE/C370, shows a comprehensive health equity content report from the FLO25 system.

**Figure 2.**
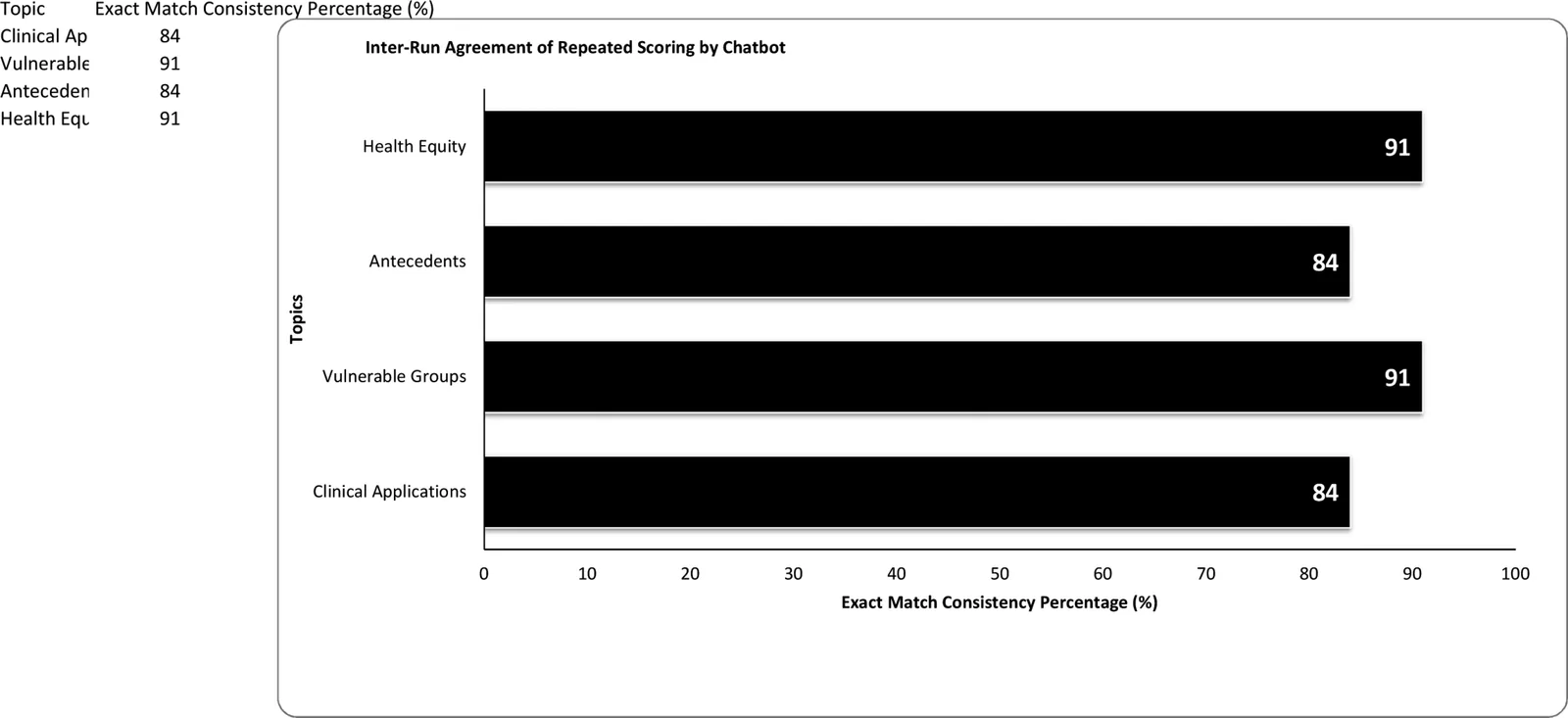
Interrun Agreement of Repeated Scoring by Chatbot.

### Validity Testing With Faculty Raters

Figure 3 presents the proportion of agreement between the chatbot’s modal scores and faculty modal ratings across the 4 rubric topics. Faculty-chatbot agreement was highest for Health Equity coverage (89%), indicating that the model reliably identified whether core health equity concepts were present in the lecture slides. Faculty-chatbot agreement was moderate for Antecedents (56%) and Vulnerable Groups (56%), reflecting partial alignment in detecting content related to structural determinants or marginalized populations. For Clinical Applications, agreement rates were 67%, showing that the chatbot could often but not always identify whether the materials provided actionable or practice-oriented equity guidance. Together, these results demonstrate substantial but topic-specific variability in agreement with faculty ratings.

**Figure 3.**
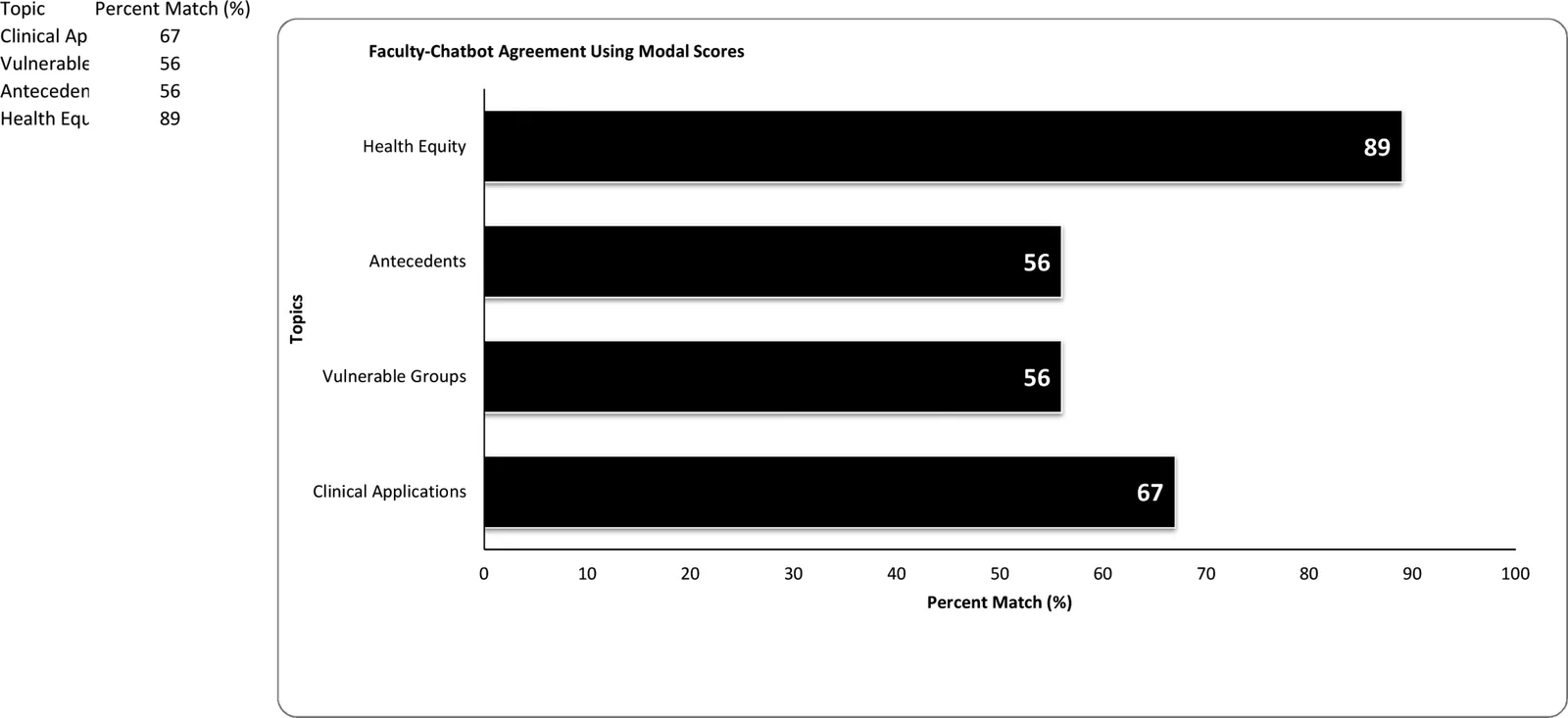
Faculty-Chatbot Agreement Using Modal Scores.

### Internal Focus Group Regarding Utility

The focus group discussion included insights from one midwifery faculty member who designed the course material and 2 independent midwifery faculty reviewers who were not part of the course or the course development. They all served as “human raters” for this study. While the chatbot demonstrated the ability to provide detailed and nuanced feedback, the team emphasized that it was not a replacement for human evaluation.

Several potential applications of AI were identified, including mapping the curriculum, aligning course materials with accreditation standards, and evaluating health equity coverage across lectures. The participants felt that chatbot tools provided value through their ability to provide consistent, detailed, and nonjudgmental feedback that goes beyond simple scoring. They felt the chatbot offered descriptive analyses that can help educators understand where health equity concepts are addressed, potentially across an entire curriculum. Overall, the team emphasized the technology’s limitations and the critical role of human expertise. There was consensus about both the promising potential of and significant challenges to using AI for educational content analysis.

### Limitations

This analysis was conducted at one institution and in one advanced practice nursing specialty. The evaluated course content was PowerPoint slides that did not include full transcripts or supplemental content. Additionally, the chatbot could not assess the content of images, nor capture classroom discussions. Limitations in sample size and sample type necessitate further testing in different environments and with more users to assess transferability and the value of outputs. Additional research is needed to understand the impact of chatbot-based feedback on educators and their teaching practices.

## Conclusions

There are significant challenges to using LLMs for evaluation work, including potential bias, difficulty capturing contextual nuances, and the need for continuous refinement. It is worth noting that the FLO25 chatbot was viewed as a supplemental tool, not a replacement for human evaluation. The importance of interdisciplinary collaboration in developing this AI tool and workflow cannot be understated. Creating a reliable chatbot required extensive work in defining instructions, validating outputs, and understanding the specific domain of application.

While this pilot study only used nurse-midwifery course content from one course, the rating approach is applicable for the assessment of health equity content beyond this specific course and specialty area. This type of automated content review has applications in curricular assessment and evaluation processes, including the expansion of rubrics beyond health equity. To ensure effectiveness, evaluators must clearly define the goals of the content analysis. It is important to determine the acceptable range of reliability and necessary validation required for different applications.

In this case, chatbot feedback was intended for faculty reflection. This may require less precision because it will be utilized by a human expert who can determine quality, accuracy, and applicability. Chatbot analysis that will be used without expert human interpretation, for example, provided directly to students, requires more precision.

This study highlights potential applications of using RAG to supplement curriculum evaluation and continuous quality improvement in nursing education. Accreditation and faculty development activities could also benefit from this approach.^25^ In addition, RAG-enabled chatbots can analyze content beyond PowerPoint slides, including recordings of presentations, lecture notes, course outlines, syllabi, administrative documents, and research materials.

This study used multiple methods to test the reliability, validity, and utility of a custom chatbot in providing automated feedback on health equity content in teaching presentations. The project underscored that while AI has potential in educational settings, it currently requires substantial human oversight and interpretation to be valuable. At the moment, chatbot feedback can be helpful for educator reflection, but automated scoring should not be used as definitive in an evaluation context.

## Supplementary Material


