# Supplementary material for: Artificial Intelligence Enhanced Content Review for Curriculum Evaluation in Nursing Education: A Pilot Study
Source: Nurse Educ. 2026 Jul 9;51(6):311–6. doi: 10.1097/NNE.0000000000002243 (PMC13693971; doi:10.1097/NNE.0000000000002243)
Supplement: Supplementary file 1 [file nedu-51-0311-s001.pdf]

Appendix A: Health Equity Content Rubric

Faculty Lecturer:	
Lecture Title:	
Lecture Date:	
Source: (powerpoint slides without audio were uploaded)	

Content Topic	Low	Medium	High
Coverage of Health Equity	Minimal or no definition of health equity and social determinants of health	Provides a basic definition of health equity and social determinants of health	Comprehensive coverage of health equity concepts, including clear definition and discussion of social determinants of health
Coverage of Health Equity Antecedents	Mentions health equity antecedents superficially or not at all; no clear connection to	Introduces one or more antecedents with brief or generalized explanations; limited integration	Demonstrates in-depth understanding of one or more health equity antecedents with thorough explanation and clear connection to

	health equity concepts	into the broader discussion of health equity	health equity concepts and frameworks
Addressing Vulnerable Groups	No clear definition of vulnerable groups	Provides a definition of vulnerable groups, but does not discuss barriers they face	Clearly defines vulnerable groups and discusses the barriers they face in accessing health care
Application of Health Equity in Clinical Practice	No resources or steps provided for applying health equity in clinical practice	Provides some resources or steps for applying health equity in clinical practice	Provides clear, comprehensive resources and steps for applying health equity principles in clinical practice

Appendix B: Example output

Health Equity Lecture Review: Contraception in the Postnatal Period

Metadata

Faculty Lecturer: [Redacted]

Lecture Title: Contraception in the Postnatal Period

Lecture Date: 2025

Source: 3_Postpartum_Contraception.pptx (lecture slide set, no audio)

Key Points

1. Presents comprehensive coverage of various contraceptive options for the postpartum period, including efficacy, mechanisms of action, advantages, and disadvantages
2. Explicitly addresses health equity concerns related to contraception access and historical reproductive injustice
3. Discusses barriers to contraception uptake including cost, insurance coverage, healthcare inequities, and restrictive policies
4. Introduces the concept of reproductive justice as a framework for contraceptive counseling
5. Examines the compatibility of various contraceptive methods with lactation/breastfeeding
6. Highlights the importance of person-centered contraceptive counseling approaches

Health Equity Evaluation

1. Coverage of Health Equity Concepts: HIGH

The lecture provides comprehensive coverage of health equity concepts related to contraception, including: - Explicit discussion of healthcare inequities as barriers to contraception uptake [Slide 4] - Acknowledgment of historical reproductive injustices including forced sterilization [Slide 5] - Clear presentation of reproductive justice as a framework [Slide 7] - Recognition of disproportionate targeting of marginalized groups for contraceptive coercion [Slide 5] - Detailed section on health equity key points [Slide 6]

The lecturer states: "Contraceptive Counseling should be person-centered and follow the principles of reproductive justice" and "Providers must be aware of their own biases and internalized racism when counseling clients" [Slide 6].

2. Coverage of Health Equity Antecedents: MEDIUM

The lecture addresses several antecedents to health equity in contraception: - Financial/Economic factors: "Cost and Insurance Coverage" as barriers [Slide 4] Law/Policy elements: "Restrictive Legal and Legislative Climate" affecting contraceptive access [Slide 4] Societal structures: "Medicaid expansion for 1 year" versus "traditional = 60 days" [Slide 4] However, the lecture could more deeply explore how these antecedents systematically create disparities in contraceptive access and outcomes across different populations.

3. Addressing Vulnerable Groups: HIGH

The lecture clearly identifies and discusses vulnerable populations regarding contraceptive access and coercion: - Disproportionately target individuals who are: Poor, Young, Incarcerated, Or members of communities of color, Those with disabilities" [Slide 5] - Discusses specific barriers for "birthing families who rely on Medicaid" [Slide 6] - Addresses needs of trans and nonbinary individuals [Slide 13]: "for individuals at risk for pregnancy there are NO contraindications for any type of contraception while on Testosterone" - Notes historical targeting of "groups deemed undesirable to reproduce" [Slide 6] - Includes discussion of Kentucky's historical involvement in eugenics [Slide 7 notes]

4. Application of Health Equity in Clinical Practice: HIGH

The lecture provides clear, practical guidance for applying health equity principles in contraceptive counseling: - Outlines strategies to address barriers: "Expansion of Medicaid through 1 year PP, Immediate LARC insertion, Telehealth, Optimizing PP care with an initial visit in the first 3 wks PP" [Slide 6] - Emphasizes client-centered approach to contraceptive counseling [Slide 8-10] - Provides specific questions for counseling that respect autonomy [Slide 10] - Warns against potential for coercive counseling with immediate postpartum LARC: "Recognize bias!! could lead to coercive counseling to people of color, encouraging them to use a LARC method that may not fit their personal preferences or values" [Slide 85] - Encourages awareness of provider biases: "Providers must be aware of their own biases and internalized racism when counseling clients" [Slide 6]

Summary Assessment

This lecture provides an excellent integration of health equity principles within postpartum contraception education. The presenter effectively balances clinical information with historical context and social justice frameworks. Particularly strong is the explicit acknowledgment of reproductive coercion history and the emphasis on person-centered counseling that respects autonomy.

The lecture's discussions of reproductive justice, contraceptive coercion, and strategies to address healthcare inequities demonstrate a commitment to preparing healthcare providers to deliver equitable contraceptive care. By highlighting biases in contraceptive counseling and specifically warning against coercive practices, the lecturer encourages reflective practice among healthcare providers.

While the lecture touches on various antecedents to health inequity in contraceptive access, this area could be strengthened with more explicit connections between systemic issues and resulting disparities in outcomes among different populations.

Overall, this lecture serves as a strong example of how to integrate health equity content into clinical education on contraceptive management.

Disclaimer: This content is produced by Future FLO 25 at VUSN. Always double check chatbot outputs. More information on key functions and full disclaimer here: https://nursing.vanderbilt.edu/research/initiatives/future-flo/.
